# ICTV Virus Taxonomy Profile: Kolmioviridae 2024

**DOI:** 10.1099/jgv.0.001963

**Published:** 2024-02-29

**Authors:** Jens H. Kuhn, Artem Babaian, Laura M. Bergner, Paul Dény, Dieter Glebe, Masayuki Horie (堀江真行), Eugene V. Koonin, Mart Krupovic, Sofia Paraskevopoulou (Σοφία Παρασκευοπούλου), Marcos de la Peña, Teemu Smura, Jussi Hepojoki

**Affiliations:** 1 Integrated Research Facility at Fort Detrick, Frederick, Maryland, USA; 2 University of Toronto, Toronto, Canada; 3 MRC-University of Glasgow Centre for Virus Research, Glasgow, UK; 4 University Sorbonne Paris Nord, Bobigny, France; 5 Justus Liebig University, Giessen, Germany; 6 Osaka Metropolitan University, Izumisano, Osaka, Japan; 7 National Center for Biotechnology Information, Bethesda, Maryland, USA; 8 Institut Pasteur, Université Paris Cité, Archaeal Virology Unit, Paris, France; 9 Genome Competence Center (MF1), Robert Koch Institute Berlin, Berlin, Germany; 10 Universidad Politécnica de Valencia-CSIC, Valencia, Spain; 11 University of Helsinki, Helsinki, Finland

**Keywords:** deltavirus, hepatitis D virus, ICTV Report, *Kolmioviridae*, taxonomy

## Abstract

*Kolmioviridae* is a family for negative-sense RNA viruses with circular, viroid-like genomes of about 1.5–1.7 kb that are maintained in mammals, amphibians, birds, fish, insects and reptiles. Deltaviruses, for instance, can cause severe hepatitis in humans. Kolmiovirids encode delta antigen (DAg) and replicate using host-cell DNA-directed RNA polymerase II and ribozymes encoded in their genome and antigenome. They require evolutionary unrelated helper viruses to provide envelopes and incorporate helper virus proteins for infectious particle formation. This is a summary of the International Committee on Taxonomy of Viruses (ICTV) Report on the family *Kolmioviridae*, which is available at ictv.global/report/kolmioviridae.

## Virion

**Fig. 1.**
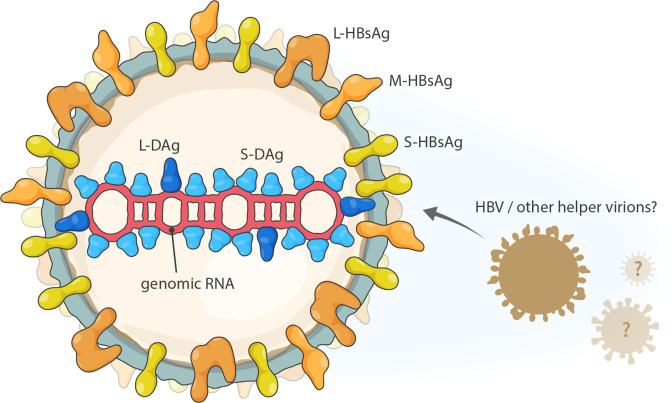
Schematic of hepatitis D virus 1 particles. L-DAg, S-DAg: large and small delta antigen; L-HBsAg, M-HBsAg, S-HBsAg: large, middle and small HBV surface antigen; HBV: hepatitis B virus.

Kolmiovirions are spherical with a lipid envelope containing envelope proteins obtained from an evolutionarily unrelated helper virus (Table 1*,* Fig. 1). Virions contain a ribonucleoprotein (RNP) complex consisting of distinct isoforms of delta antigen (DAg) that are encoded in and closely associate with deltavirus genomic and antigenomic RNAs [1,2].

**Table 1.** Characteristics of members of the family *Kolmioviridae*

Example:	hepatitis D virus 1 (AF104263), species *Deltavirus italiense*, genus *Deltavirus*
Virion	Spherical virions (36–43 nm in diameter) with an outer envelope containing envelope proteins derived from a helper virus and an inner ribonucleoprotein consisting of genomic RNA and a nucleocapsid protein (delta antigen; DAg) that is encoded by the genomic RNA and may occur in two isoforms
Genome	Rod-like ribozyme-containing negative-sense, covalently closed, circular RNA (cccRNA) of about 1.5–1.7 kb
Replication	RNA-directed RNA synthesis by host-cell DNA-directed RNA polymerase II through a double rolling circle mechanism, and autocatalytic cleavage/ligation via encoded genomic and antigenomic ribozymes and re-cyclization in the host-cell nucleus
Translation	mRNA-based translation of DAg (in some cases including an isoform thereof)
Host range	Mammals, amphibians, birds, fish, insects and reptiles
Taxonomy	Realm *Ribozyviria*; the family includes >7 genera and >14 species

## Genome

**Fig. 2.**
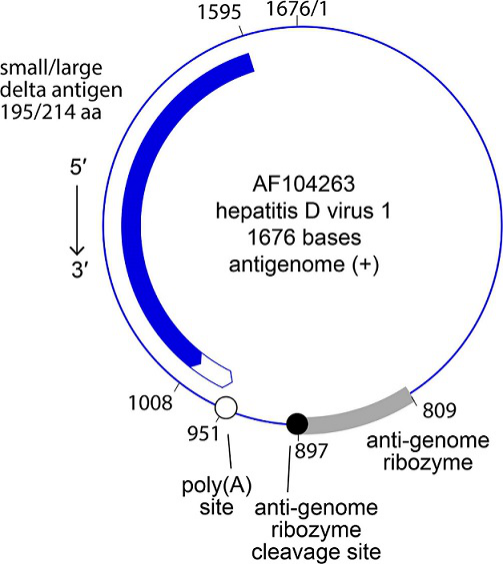
Antigenome organization of hepatitis D virus 1. Nucleotide numbering according to that for the (complementary) genome.

Kolmiovirids have a viroid-like, ribozyme-containing, negative-sense, covalently closed circular RNA (cccRNA) genome of about 1.5–1.7 kb (Fig. 2) that forms a rod-like structure through its high degree of self-complementarity. The genome encodes a single protein, DAg, which in some cases occurs in two isoforms [1,8].

## Replication

Kolmiovirion cell entry is mediated by interaction with cell-surface receptors and envelope proteins that are obtained from helper viruses. Membrane fusion releases the RNP into the cytosol, from where it migrates to the cell nucleus where RNA-directed RNA genome and antigenome synthesis is mediated by host-cell DNA-directed RNA polymerase II through a double rolling circle mechanism; genome- and antigenome-encoded ribozymes catalyse autocatalytic cleavage of concatenated progeny genomes and ligation/recyclization in the nucleus. DAg isoforms serve as nucleoproteins and regulate replication and packaging [1,2].

## Pathogenicity

Deltaviruses cause disease in humans, typically in association with the evolutionarily unrelated hepatitis B virus (HBV, family *Hepadnaviridae*). Coinfection causes hepatitis D, the most severe of WHO-classified viral hepatitides [1,2].

## Taxonomy

Current taxonomy: ictv.global/taxonomy. Kolmiovirids (realm *Ribozyviria*) are most closely related to viroids and related mobile genetic elements. Kolmiovirids share at least two of the following characteristics: (i) enveloped virions; (ii) circular negative-sense RNA genome containing ribozymes and encoding a DAg homolog; (iii) require an evolutionarily unrelated helper virus for assembly of infectious virions.

## Resources

Full ICTV Report on the family *Kolmioviridae*: www.ictv.global/report/kolmioviridae.

## Abbreviations

cccRNA: covalently closed, circular RNA
DAg: delta antigen
HBV: hepatitis B virus
L-DAg, S-DAg: large and small delta antigen
L-HBsAg, M-HBsAg, S-HBsAg: large, middle and small hepatitis B virus surface antigen
RNP: ribonucleoprotein
